# Multipoint Fiber Loop Ringdown Sensors for Large Strain Measurement Using Frequency-Shifted Interferometry

**DOI:** 10.3390/s19132907

**Published:** 2019-07-01

**Authors:** Chunfu Cheng, Zehao Chen, Yiwen Ou, Jiaxuan Chen

**Affiliations:** 1 Hubei Collaborative Innovation Center for High-Efficiency Utilization of Solar Energy, Hubei University of Technology, Wuhan 430068, China; 2 School of Science, Hubei University of Technology, Wuhan 430068, China

**Keywords:** fiber loop ringdown, frequency-shifted interferometry, strain sensor, large measuring range

## Abstract

A novel multipoint fiber loop ringdown (FLRD) strain sensing system using frequency-shifted interferometry (FSI) is proposed and experimentally validated. Compared to conventional multipoint FLRD techniques, this scheme measures the decay rate of the continuous wave (CW) light in the space domain and thus greatly reduces the cost without the requirement of expensive devices. A serial dual-point strain sensing system was experimentally constructed and a biconical tapered multimode fiber (MMF) as the sensor head was used for obtaining the large measuring range. By applying different strains on the sensor heads through translation stages, a linear response between strain and additional loss induced by strain sensor was obtained, and the static strain sensitivities of 0.13676 dB/mε and 0.19665 dB/mε were achieved, corresponding to the detection limit of 0.0123 dB and 0.0360 dB, respectively. Moreover, a large measuring range of approximately 6 mε was achieved for both strain sensors. The experimental results indicate that our proposed method offers a promising multipoint strain sensor which has the advantages of low cost, a simple sensing structure and a large measuring range.

## 1. Introduction 

For the past decades, multipoint fiber-optic strain sensors have played an increasingly significant role in structural health monitoring due to the advantages of low cost, high sensitivity, large multiplexing capability, immunity to electromagnetic fields and robustness in hazardous environments [1,2,3,4]. A variety of multipoint fiber-optic strains sensing techniques have been developed, including time-division multiplexing (TDM) [5], wavelength division multiplexing (WDM) [6] and the frequency-modulated continuous-wave (FMCW) technique [7]. In the TDM scheme, in order to distinguish each strain sensor, a fast optical switch is needed to extract only one pulse from the pulse trains reflected by the fiber Bragg grating (FBG) array. Therefore, the measurement time will increase as the number of strain sensors increases, that is to say, the measurement points are limited by the measurement time in the TDM scheme. Unlike TDM method, the WDM scheme distinguishes each FBG-based strain sensor by measuring the reflection wavelengths of the FBG array using an optical spectral analyzer (OSA), which can enhance the sensing capacity. However, the measuring range is limited by the bandwidth of the light source. In order to improve the measuring range, recently Jingjing Guo et al. proposed multi-point strain measurements by using coherent dual-comb pulses with a broad bandwidth of 1.2 THz. Using this method, a large measuring range of 520 με was achieved in monitoring five cascaded FBG strain sensors [8]. In 2017 Atsushi Wada et al. proposed multi-point strain measurements by using the Fabry–Perot interferometer (FPI) and the measuring range of approximately 700 με was achieved [9]. However, the measuring range is still limited, especially in the strain measurement for concrete structures, in which the maximum strain usually can reach 5 mε or larger. Furthermore, the measurement point used by WDM is limited to a few in such large strain measurements. Compared to the TDM technique, the FMCW technique can improve the signal-to-noise ratio (SNR), but it needs modulation on the light source, and thus the multipoint strain sensing system is very complicated. For all of the above mentioned methods, usually the FBG-based strain sensor is used and the sensitivity is ultimately limited by the limited spectral resolution of the OSA, which also makes the sensing system too expensive. To avoid using high spectral resolution OSA due to the cost, recently Aitor Lopez-Aldaba et al. used the microstructured optical fibers as the sensor heads by monitoring their fast Fourier transform phase variations [10]. The results showed a sensitivity of 0.00059 πrad/με with a measuring range of 450 με was achieved, the cost was reduced, but the sensitivity and measuring range were still limited. Therefore, cost effective and highly sensitive multipoint strain sensors with large measuring ranges are still desired in large strain measurement of concrete structures. 

In recent years, the fiber loop ringdown (FLRD) technique has been employed in fiber sensor technology [11,12,13,14,15,16]. This technique measures the decay rate (ringdown time) of the optical pulse in time domain rather than the intensity decay of the laser source. Therefore, the sensing stability is improved due to its immunity to light source fluctuation. Furthermore, the detection sensitivity is greatly enhanced due to its multiple-pass enhanced detection method. Due to the above advantages, FLRD has been widely used in the single-point measurement of physical or chemical parameters, such as strain [12,13,14], pressure, gas concentration, and so on. In order to meet the demands for multipoint strain sensing, several multipoint FLRD strain sensing methods have emerged over the years [15,16]. The strain detection limit is better than the above mentioned methods. To obtain the ringdown signal for the FLRD-based strain sensing scheme, a pulsed laser and a high-speed detector are needed. Thus, the cost is high which limits the use of this technique in real applications. 

In 2011, a FLRD based on frequency-shifted interferometry (FSI) has been proposed by the group of Li Qian. Compared with FLRD schemes, FSI-FLRD technique uses a CW laser source, slow detector and low-speed data acquisition to observe the ringdown signal [17], which greatly reduces the cost. It measures the changes in the decay rate of the CW light in the space domain instead of the decay rate of an optical pulse in the time domain. Therefore, FSI-FLRD belongs to the space domain FLRD technique. In addition, differential detection is used to eliminate the direct current (DC) noise of the interference signal and thus the SNR is further improved, which provides the sensing system with high stability. Since its advent, it has been used in magnetic field [18], pressure [19], and gas concentration detection [20,21,22]. However, no reports have been found on strain sensing using FSI-FLRD. 

In this paper, a cost-effective multipoint strain sensing system with a large measuring range based on FSI-FLRD was proposed. Here, to reduce the cost, ease of fabrication and achieve the large measuring range, a single-mode–multimode–single-mode (SMS) structure was used for large strain sensing. A dual-point FSI-FLRD strain sensing system was experimentally constructed to characterize the proposed system. By measuring the additional loss of the two strain sensors under different applied strains, high strain sensitivities of 0.13676 dB/mε and 0.19665 dB/mε were observed respectively, and the corresponding minimum detectable losses of 0.0123 dB and 0.0360 dB were obtained. Furthermore, a large measuring range of 6 mε for both strain sensors was achieved. This is larger than that previously reported for strain sensors [9,10]. To the best of the authors’ knowledge, this is the first time that FSI-FLRD has been used to detect multipoint strains. The experimental results showed that the proposed sensing system was capable of performing multipoint strain measurements with a large measuring range, good linear responses, simple sensing structures and low costs.

## 2. Sensing Configuration and Principle

**Figure 1:**
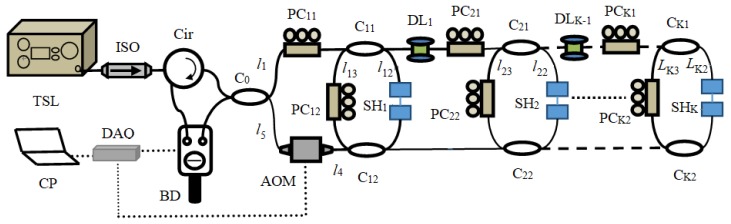
Schematic of the FSI-FLRD multipoint strain sensing system. TSL: tunable semiconductor laser; ISO: isolator; Cir: circulator; C_0_: 3 dB fiber coupler; C_i1_, C_i2_: 99.5/0.5 fiber couplers; PC_i1_, PC_i2_: polarization controller; SH_i_: sensor head; DL_i_: fiber delay line; AOM: acousto-optic modulator; BD: balanced detector; DAQ: data acquisition card; CP: Computer.

The experimental setup of the multipoint strain sensing system based on FSI-FLRD is depicted in Figure 1. In this system, each FLRD cavity is composed of two couplers by connecting their output ports. As shown in Figure 1, multiple FLRD cavities are cascade connected and embedded in a frequency-shifted Sagnac interferometer [17]. A tunable semiconductor laser (TSL) is used as the CW source. After passing through the isolator, circulator and fiber coupler C_0_, the laser beam is split into two counter-propagating lightwaves and starts to circulate in opposite directions in the FLRD cavities one by one. A small portion of the light exits from each FLRD cavity every time the light completes the same number of trips and then it returns to the coupler C_0_. If the cavity length is much longer than the coherence length of the light source, the two lightwaves interfere at the coupler *C*_0_ and then the interference signal is detected by a balanced detector (BD). According to the FSI-FLRD theory described in Ref. [17], the differential interference signal Δ*I* can be expressed as follows.

(1) $$\Delta I=\sum_{i=1}^{K}\sum_{m=0}^{\infty }I_{im}\cos[2\pi \frac{n(mL_{i}+L_{i0})}{c}f]=\sum_{i=1}^{K}\sum_{m=0}^{\infty }I_{im}\cos[2\pi F_{im}f]$$

where *K* is the total number of FLRD cavity, *L_i_ = l_i_*_2_ + *l_i_*_3_ (*i* = 1, 2, …, *K*) is defined as cavity length for the *i*th FLRD cavity and *l_i_*_2_ and *l_i_*_3_ are defined as the fiber lengths shown in Figure 1. *L_i_*_0_ = *l*_1_ + *l_i_*_2_ + *l*_4_ − *l*_5_ + *d_i_*_−1_ is a length constant, and *d_i_*_−1_ is the length of the (*i* − 1)th optical fiber delay line, *F_im_ = n(mL_i_ + L_i_*_0_)/*c* is the oscillation frequency of the *i*th FLRD cavity where *m* is the number of cavity roundtrips traveled by the light, *n* is the effective refractive index of the fiber core, *c* is the speed of light in vacuum. Note that *c* is a very large value and thus the *F_im_* is very small, which means the sensing system operates at a low frequency. Therefore, the slow detection is realized in the FSI-FLRD sensing system. *f* is the frequency shift induced by the acousto-optic modulator (AOM). *I_im_* is the intensity of the interferential light coming from the *i*th FLRD cavity, which obeys the Beer-Lambert’s law:

(2) $$I_{im}=I_{i0}\cdot \exp(-m\alpha_{ic}/4.34)=I_{i0}\cdot \exp(-\frac{l_{i}}{4.34L_{i}}\alpha_{ic})$$

where *I_i_*_0_ is the initial light intensity, *l_i_* = *mL_i_* is the distance traveled by the light in the *i*th FLRD cavity, and *α_ic_* is the empty cavity loss in decibels when the strain is not loaded on the sensor head. 

After performing a fast Fourier transform (FFT) on the interference signal Δ*I*, the FLRD decay signal in the space domain can be obtained. When the amplitude of the space domain Fourier spectrum decreases to 1/e of its initial intensity, the corresponding distance is defined as the ringdown distance. According to Equation (2), the ringdown distance *d_i_*_0_ for the FLRD cavity without strain applied usually called empty cavity is:

(3) $$d_{i0}=\frac{4.34L_{i}}{\alpha_{ic}}$$

When the strain is loaded in the sensor head, the additional loss *α_is_* (in decibels) induced by the strain sensor will cause a change in the ringdown distance *d_i_*, which has the following relationship with *α_is_*:

(4) $$d_{i}=\frac{4.34L_{i}}{\alpha_{ic}+\alpha_{is}}$$

The additional loss of each strain sensor in decibels will be derived based on Equations (3) and (4):

(5) $$\alpha_{is}=4.34L_{i}(\frac{1}{d_{i}}-\frac{1}{d_{i0}})$$

In this paper, a section of multimode fiber (MMF) is used as the strain sensor head. Suppose *L_f_* is the MMF length between the two fixing points at the two translational stages, Δ*L_f_* is the changed length caused by the moving translational stage, and then the strain ε on the MMF can be detected by measuring Δ*L_f_* and *L_f_* due to their relationship satisfied by $\varepsilon =\Delta L_{f}/L_{f}$ [13,14]. Then, the additional loss $\alpha_{is}$ induced by the *i*th strain sensor can be further given by [13,14]:

(6) $$\alpha_{is}=k\varepsilon =k\frac{\Delta L_{if}}{L_{if}}$$

where k is the strain induced attenuation coefficient. Equation (6) shows that increasing the length (*L_if_*) of the sensing fiber or reducing the length change (Δ*L_if_*) can reduce the additional loss of the sensor head, and thus improve the sensitivity. However, a higher sensitivity gives a smaller measuring range. Therefore, the length change should not be too small to obtain the large measuring range. For a given length change, the sensitivity can be enhanced simply by increasing the length of the sensing fiber.

The detection limit is often characterized by the minimum detectable additional loss induced by the strain sensor, which can be written as [13]:

(7) $$\alpha_{is}^{\min}=\frac{4.34}{h}\frac{\delta d_{i}}{{\bar{d}}_{i}}$$

where $h=d_{i0}/L_{i}$, $\delta d_{i}$ is the standard deviation of the ringdown distance, ${\bar{d}}_{i}$ is the mean ringdown distance. Equation (7) shows that the detection limit is determined by the baseline stability of the ringdown distance $\delta_{d_{i}}/{\bar{d}}_{i}$ and the number of roundtrips *h*. In the FSI-FLRD technique, differential detection is used for improving the SNR and stability, and also the ringdown distance is immune to the power fluctuation of CW light source, therefore, FSI-FLRD has better baseline stability and a higher detection limit under the same condition compared with the conventional FLRD techniques [19].

## 3. Experimental Results and Discussion

**Figure 2:**
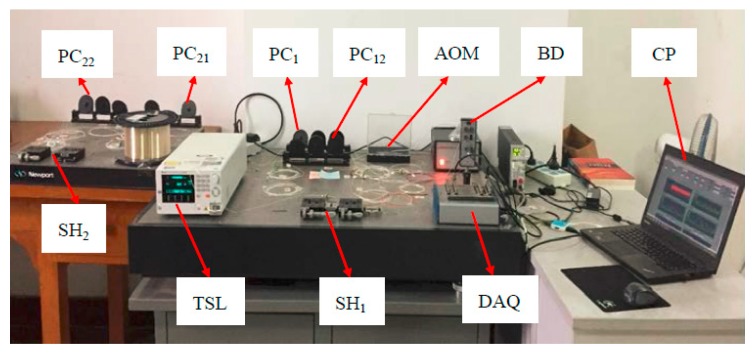
The experimental setup of the dual-point strain FSI-FLRD detection system. TSL: tunable semiconductor laser; PC_i1_, PC_i2_: polarization controller; SH_i_: sensor head; AOM: acousto-optic modulator; BD: balanced detector; DAQ: data acquisition card; CP: Computer.

The experimental setup of the dual-point strain detection system using FSI-FLRD is shown in Figure 2. It consisted of a TSL (Santec, TSL-550C) as the CW light source, an AOM as the frequency shifter, BD for differential detection, multiple polarization controllers for improving the visibility of the interference fringes, a data acquisition card (DAQ) and so forth. The output power of TLS was set to 8 mW at 1550 nm. A ~2.5 km fiber delay line was used to avoid the interference of the ringdown signals from the different FLRD cavities. The AOM was swept from 90 MHz to 110 MHz at steps of 0.02 MHz with frequency hopping time of 1 ms, which was synchronized with the DAQ. The sampling rate of the DAQ was set to only 100 kS/s, which was much lower than that of the traditional FLRD techniques [12,13,14]. A LabVIEW program was developed for real-time data processing.

**Figure 3:**
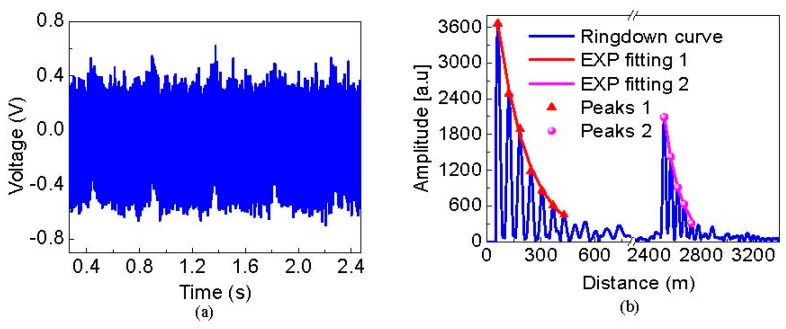
The typical differential interference signal without strain applied. (**a**) The time-domain signal sampled by the DAQ; (**b**) the space-domain ringdown signal obtained by performing fast Fourier transform of the time-domain signal. 1 represents sensing point one, 2 represents sensing point two.

**Figure 4:**
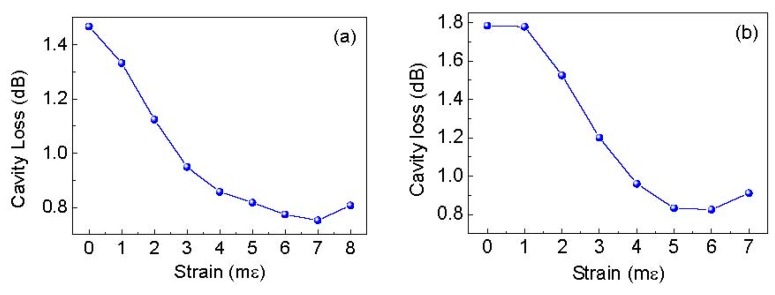
Cavity loss as a function of strain when the multimode fiber (MMF) is drawn into the tapered fiber. (**a**) Sensing point one, (**b**) sensing point two.

The SMS strain sensor head was composed by a graded-index MMF with a length of 20 cm between two standard single-mode fibers. It was fabricated by using a precision Fujikura CT-07 cleaver and a Fujikura 62 C fusion splicer. Following this, a fraction of MMF of the sensor head was glued on two translation stages (Newport M-423) using AB German adhesive glue (xuanxin 1016). The distance between the two glued points was ~5 cm, and the MMF was stretched by the translation stages at a step of 0.05 mm. Note here the buffer layer of MMF was not stripped, for it had to be verified that MMF was suitable for strain measurements with a large measuring range and lower temperature cross sensitivity than that of FBG-based or FP-based strain sensors [23,24]. However, the SMS-based strain sensor causes large empty cavity loss because of the mode mismatch between MMF and SMF, and the large empty cavity loss leads to low sensitivity. As a result, low empty cavity loss is required to enhance the sensitivity. Fortunately, Peter B. Tarsa [12] reported that empty cavity loss can be lowered by using a biconical tapered MMF, because the biconical MMF taper can induce the excitation of low-order cladding modes, reconvert them to propagating core modes and improve the coupling efficiency of the light into the SMF. Therefore, to improve the sensitivity, the tapered MMF is a better choice. Usually the tapered MMF is fabricated by heating and drawing technology [25]. As the buffer layer of MMF is un-stripped for large strain measurement, here the tapered MMF is fabricated only by drawing technology of the translation stages. To obtain the smallest empty cavity loss, the authors slowly stretched the MMF and monitored the change of the cavity loss of the FSI-FLRD system in real time. Figure 3a shows a typical time-domain differential interference signal measured by FSI-FLRD when the strain was not applied. Performing FFT on the time-domain differential interference signal, two clear ringdown signals in the space domain can be observed in Figure 3b. It was noted that a Hann window was applied in the FFT process for reducing the sidelobe crosstalk of the Fourier peaks. Zero padding was also used in the FFT process with an FFT size of 2^20^ for improving the location accuracy of the Fourier peaks. Using the peak-picking algorithm, the Fourier peaks were found, and then the exponential (EXP) decay curves were obtained by fitting these Fourier peaks with a simple first order EXP function. According to the definition of ringdown distance, it was calculated out to be 181.56 m for the first sensing point and 127.15 m for the second sensing point. The cavity lengths for the two FLRD cavities were approximately 61.38 m and 52.28 m respectively, by subtracting the locations of the two adjacent Fourier peaks. According to Equation (3), the corresponding empty cavity losses were estimated to be 1.4672 dB and 1.7844 dB. Using the same methods mentioned above, the relationship between cavity loss and the strain applied on the SMS sensor head was achieved as shown in Figure 4. As it can be seen from Figure 4, there is an optimal strain which is corresponding to the lowest cavity loss. The main reasons are as follows: Firstly, the MMF becomes thinner and thinner until it forms a biconical tapered fiber when the applied strain increases. In this tapered stage, mode excitation occurs in the MMF, low-order cladding modes are converted to propagating core modes [12], and more light power enters into the fiber core of the MMF. Therefore, it results in the reduction of the cavity loss. At this moment, when the strain is further increased, the core modes are reversely converted to cladding modes, the light power in fiber core is reduced and thus the cavity loss increases. Hereto, two biconical SMS strain sensors were fabricated. For the sensing point 1, the lowest cavity loss was 0.7523 dB at the optimal strain of 7 mε, whereas for the sensing point 2, it was 0.8254 dB corresponding to the optimal strain of 6 mε. 

**Figure 5:**
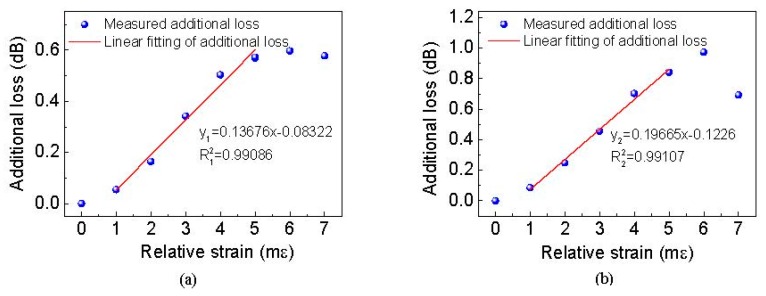
Additional loss induced by strain sensor as a function of relative strain. (**a**) Sensing point one, (**b**) sensing point two.

This slight difference may be caused by the different fusion splice losses, coupler additional loss and polarization drifts [26]. Therefore, the authors chose the optimal strain of 7 mε and 6 mε as the initial strain for the sensing points 1 and 2, respectively. Then, using the FSI-FLRD technique, the additional losses were measured as shown in Figure 5 when the relative strain increased from 0 to 7 m*ε* at an increment of 1 m*ε*. When the applied relative strain was less than 6 m*ε*, the additional loss induced by the strain sensor increased at a near-linear trend for both sensing points, especially in the region from 1 mε to 5 mε, the R-squares of both strain sensors were larger than 0.99, which indicated the strain sensing system had a good linear response. The linear relationship is also consistent with the theoretical expectations of Equation (6). The measurement strain sensitivity, that is, the slope of the linear fitting curve is 0.13676 dB/m*ε* for sensing point 1 and 0.19665 dB/m*ε* for sensing point 2. Moreover, according to Equation (6), lower additional loss induced by the strain can be obtained by increasing the length of sensing fiber (*L_if_*). The lower additional loss will result in the higher sensitivity, so the sensitivity of our proposed strain sensor can be further improved by using a longer sensor length. When the applied strain was larger than 6 mε, the response of the two strain sensors began to deviate from the linear trend due to the fact that the strain exceeded the tolerance of the SMS sensor. Therefore, the measuring ranges are 0–6 mε for both strain sensors, which is larger than those of typical FBG or FP-based sensors [8,9,27]. Moreover, the FSI-FLRD strain sensor never needs use of the expensive high spectral resolution OSA. Therefore, the proposed scheme has the advantages of large measuring ranges, high sensitivity and low cost, and it is very suitable for the practical applications that need the large strain measurement.

**Figure 6:**
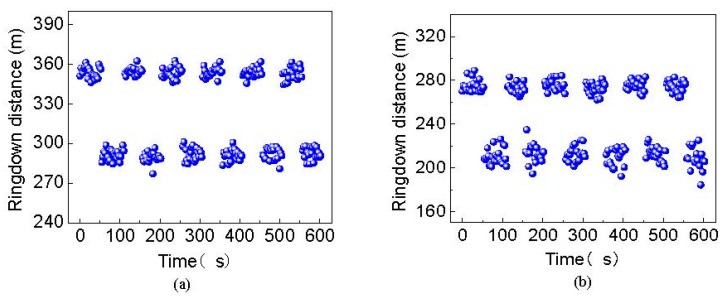
The repeated measurement results with relative strain of 2 mε unloaded and loaded for (**a**) the sensing point 1 and (**b**) the sensing point 2.

**Figure 7:**
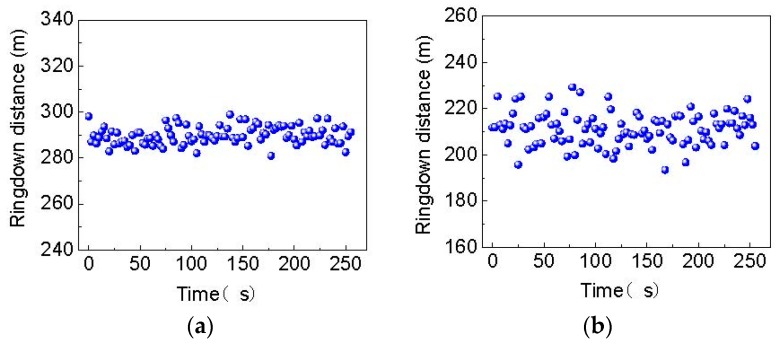
The stability curve of the strain sensing system with relative strain of 2 mε loaded for (**a**) the sensing point 1 and (**b**) the sensing point 2.

In order to evaluate the reproducibility of the FSI-FLRD strain sensors, the strain measurement was repeated under the same conditions, and the results are illustrated in Figure 6. The response of the strain sensing system is reproducible when the strain is loaded or unloaded on both sensing points, which indicates the strain sensors have a good repeatability and a rapid response. The system stability was also tested as shown in Figure 7 when the relative strain of 2 m*ε* was applied on the sensor heads. The average ringdown distances (${\bar{d}}_{i}$) for the two sensing points were separately 289.8654 m and 211.2820 m, and the corresponding standard deviations ($\delta d_{i}$) were 3.8771 m for the sensing point 1 and 7.0746 m for the sensing point 2. Therefore, the baseline stabilities $\left(\delta d_{i}/{\bar{d}}_{i}\right)$ of the two sensing points were 1.34% and 3.35%. The better stability of sensing point 1 is mainly because its initial cavity loss is smaller than that of sensing point 2. Compared with the baseline stabilities of ~1% reported in Ref. [28,29], the sensing system has a little worse stability. This is also due to its larger initial cavity loss. The baseline stabilities can be improved by choosing fiber couplers with lower insertion loss and lower coupling loss for ringdown cavities, or improving the technique of sticking a MMF sensor head on translation stages to reduce the fiber microbending loss. However, it is noted that in this paper, a large measuring range is what the authors pursued, while a large measuring range leads to a large cavity loss, and thus results in a poor stability. That is, a poor stability is not caused by the FSI-FLRD technique itself. Actually, it has been verified that the FSI-FLRD can obtain better stability [19] because the differential detection and common-path interference have been employed to eliminate the impact of external disturbances in this technique. According to Equation (7), the calculated detection limits for the two sensing points were 0.0123 dB and 0.0360 dB, respectively. This small difference between them is also caused by the different initial cavity loss. Then, from Equations (6) and (7), the minimum detectable strains for sensing point 1 and sensing point 2 were separately 0.0899 mε and 0.1831 mε. This is worse than the conventional MMF-based FLRD strain sensor in [30]. However, the proposed sensor has a much higher measuring range (6 mε) than the sensor in [30] (only 0.8 mε). It should be noted, according to [13] and Equation (6) and Equation (7), the detection sensitivities of the multipoint FSI-FLRD strain sensor can be further increased by simply increasing the length of the sensing MMF (*L_if_*), but the measuring range and the detection limit may be compromised. Therefore, by adjusting the length of the sensing MMF, the detection sensitivity, measuring range and detection limit of the sensing system can be tailored.

## 4. Conclusions

In this paper, a novel multipoint strain sensing system based on FSI-FLRD was proposed and experimentally demonstrated. While different from the time-domain FLRD techniques, FSI-FLRD is a space-domain FLRD, which greatly reduces the cost because it only needs inexpensive apparatuses such as a CW light source, an optical frequency shifter, a slow detector, and a low-speed data acquisition device. A SMS structure after tapering was used as the strain sensor head for obtaining the large measuring range and improving the sensitivity. A serial dual-point strain sensing system was investigated experimentally. This study achieved a large measuring range of ~6 mε for both strain sensors, and the strain sensitivities of 0.13676 dB/mε for sensing point 1 and 0.19665 dB/mε for sensing point 2 were obtained. Moreover, the sensitivity could be further improved by increasing the sensing MMF length. The repeatability and stability of the sensing system were also tested. The obtained detection limits of the two sensing points were 0.0123 dB and 0.0360 dB, respectively. The experimental results indicate that the proposed FSI-FLRD is capable of realizing multipoint strain sensing with the advantages of low cost, simple structures, good linear responses, a large measuring range and good repeatability. It is very suitable for use in the multipoint strain analysis of concrete structures.

## References

[1] Adewuyi A.P., Wu Z.S. (2011). Modal macro-strain flexibility methods for damage localization in flexural structures using long-gage FBG sensors. Struct. Control Health Monit..

[2] Nishiyama M., Igawa H., Kasai T., Watanabe N. (2015). Distributed strain measurement based on long-gauge FBG and delayed transmission/reflection ratiometric reflectometry for dynamic structural deformation monitoring. Appl. Opt..

[3] Takeya H., Ozaki T., Takeda N. (2015). Structural health monitoring of advanced grid structure using multipoint FBG sensors. Proc. SPIE.

[4] Chiang Y.J., Wang L., Liu W., Hsiao C.S. (2006). Temperature-insensitive multipoint strain-sensing system based on fiber Bragg gratings and optical power detection scheme. IEEE Sens. J..

[5] Chen J., Liu Q., Fan X., He Z. (2016). Sub-nano-strain multiplexed fiber optic sensor array for quasi-static strain measurement. IEEE Photonics Technol. Lett..

[6] Yeh C., Zhuang Y., Tsai N., Chow C. (2017). Capacity and capability enhancements of FBG sensor system by utilizing intensity and WDM detection technique. Smart Mater. Struct..

[7] Chan P.K.C., Jin W., Lau K.T., Zhou L.M., Demokan M.S. (2000). Multi-point strain measurement of composite-bonded concrete materials with a RF-band FMCW multiplexed FBG sensor array. Sens. Actuators A Phys..

[8] Guo J., Ding Y., Xiao X., Kong L., Yang C. (2018). Multiplexed static FBG strain sensors by dual-comb spectroscopy with a free running fiber laser. Opt. Express.

[9] Wada A., Tanaka S., Takahashi N. (2017). Multi-point strain measurement using Fabry–Perot interferometer consisting of low-reflective fiber Bragg grating. Jpn. J. Appl. Phys..

[10] Aldaba A.L., Auguste J., Jamier R., Roy P., Amo M.L. (2018). Simultaneous strain and temperature multipoint sensor based on microstructured optical fiber. J. Lightwave Technol..

[11] Wang C. (2009). Fiber loop ring-down—A time-domain sensing technique for multi-function fiber optic sensor platforms: Current status and design perspectives. Sensors.

[12] Tarsa P.B., Brzozowski D.M., Rabinowitz P., Lehmann K.K. (2004). Cavity ringdown strain gauge. Opt. Lett..

[13] Ghimire M., Wang C. (2017). Highly sensitive fiber loop ringdown strain sensor with low temperature sensitivity. Meas. Sci. Technol..

[14] Ghimire M., Wang C., Dixon K., Serrato M. (2018). In situ monitoring of prestressed concrete using embedded fiber loop ringdown strain sensor. Measurement.

[15] Gan J., Hao Y., Ye Q., Pan Z., Cai H., Qu R., Fang Z. (2011). High spatial resolution distributed strain sensor based on linear chirped fiber Bragg grating and fiber loop ringdown spectroscopy. Opt. Lett..

[16] Shang J., Zhang W., Wei S., Zhang H. (2012). Two-channel fiber microcavity strain sensor based on fiber loop ringdown spectroscopy technology. Microw. Opt. Technol. Lett..

[17] Ye F., Qi B., Qian L. (2011). Continuous-wave fiber cavity ringdown measurements using frequency-shifted interferometry. Opt. Lett..

[18] Tian H., Zhou C., Fan D., Ou Y., Yin D. (2014). Continuous-wave fiber cavity ring-down magnetic field sensing method based on frequency-shifted interferometry. Chin. Opt. Let..

[19] Ou Y., Cheng C., Chen Z., Yang Z., Lv H., Qian L. (2018). Continuous-wave fiber cavity ringdown pressure sensing based on frequency-shifted interferometry. Sensors.

[20] Tian H., Zhou C., Fan D., Ou Y., Tian T., Liang W., Li M. (2015). Continuous-wave frequency-shifted interferometry cavity ring-down gas sensing with differential optical absorption. IEEE Photonics J..

[21] Yang Z., Cheng C., Lv H., Chen Z., Chen J., Ou Y. (2018). Multichannel continuous-wave fiber cavity ringdown gas sensing utilizing frequency-shifted interferometry. Appl. Opt..

[22] Cheng C., Yang Z., Ou Y., Chen Z., Chen J., Lv H. (2019). Simultaneous measurement of gas composition and concentration combined fiber cavtiy ringdown and frequency-shifted interferometry. Opt. Fiber Technol..

[23] Tripathi S.M., Kumar A., Varshney R.K., Kumar Y.B.P., Marin E., Meunier J. (2009). Strain and temperature sensing characteristics of single-mode–multimode–single-mode structures. J. Lightwave Technol..

[24] Hatta A.M., Semenova Y., Wu Q., Farrell G. (2010). Strain sensor based on a pair of single-mode–multimode–single-mode fiber structures in a ratiometric power measurement scheme. Appl. Opt..

[25] Wang P., Brambilla G., Ding M., Semenova Y., Wu Q., Farrell G. (2011). High-sensitivity, evanescent field refractometric sensor based on a tapered, multimode fiber interference. Opt. Lett..

[26] Ye F. (2013). Frequency-shifted interferometry for fiber-optic sensing. Ph.D. Thesis.

[27] Zhu Y., Zhang Q., Liu G., Luo X., Han M. (2018). Fabry-perot sensor using cascaded chirped fiber-Bragg-gratings with opposite chirp directions. IEEE Photonics Technol. Lett..

[28] Yang Y., Yang L., Zhang Z., Yang J.J., Wang J.F., Zhang L., Deng X., Zhang Z.X. (2017). Fiber loop ring down for static ice pressure detection. Opt. Fiber Technol..

[29] Tarsa P.B., Rabinowitz P., Lehmann K.K. (2004). Evanescent field absorption in a passive optical fiber resonator using continuous wave cavity ring-down spectroscopy. Chem. Phys. Lett..

[30] Qiu H., Qiu Y., Chen Z., Fu B., Li G. (2008). Spectroscopy and Fiber Mode Converter Strain Measurement by Fiber-Loop Ring-Down. IEEE Sens. J..

